# Promising anti-leukemic effect of *Zataria multiflora* extract in combination with doxorubicin to combat acute lymphoblastic leukemia cells (Nalm-6) (in vitro and in silico)

**DOI:** 10.1038/s41598-022-16943-4

**Published:** 2022-07-25

**Authors:** Mahla Lashkari, Ahmad Fatemi, Hajar Mardani Valandani, Roohollah Mirzaee Khalilabadi

**Affiliations:** 1 Department of Hematology and Medical Laboratory Sciences, Faculty of Allied Medicine, Kerman University of Medical Sciences, Kerman, Iran; 2 Present Address: Cellular and Molecular Research Center, Gerash University of Medical Sciences, Gerash, Iran

**Keywords:** Biochemistry, Cancer

## Abstract

One of the heterogeneous hematologic malignancies of the lymphocyte precursors is ALL. ALL has two incidence peaks that were determined in 2–5 years children and 60 years old adults. Cardiotoxicity of chemotherapeutic drugs is one of important side effects which may occur during or after chemotherapy period. The aim of this study was to evaluate the effect of ZME, Dox, and combinations on Nalm-6 cells. In this vein, the cell viability was assessed by Trypan blue and MTT assay. Evaluation of apoptosis was also analyzed by Annexin-V/PI staining. Moreover, the expression of *Bax*, *Bcl-2*, *Bcl-xl, hTERT, c-Myc, P53,* and *P21* genes was detected by Real-Time PCR. Molecular docking as an in-silico method was performed for *Bcl-2* and *Bcl-xl* proteins as well. Our achievements indicated that ZME had dose-dependent effect on Nalm-6 cells and ZME synergistically potentiated Dox effect. The expression of *Bax*, *P53* and *P21* genes increased although the expression of *Bcl-2* genes decreased when cells treated with ZME/ Dox combination. Molecular docking showed the interactions of carvacrol and thymol in the active cavities of BCL2 and BCL-xl. Regarding to present study, ZME could be utilized as a combinatorial and potential drug for leukemic patients, which is under the treatment by Dox due to reducing the chemotherapy drug doses.

## Introduction

Acute Lymphoblastic Leukemia (ALL) is a hematologic malignant disorder originating from either T- or B-cells lymphoid precursors. B-cell accounts for 80–85% of ALL cases, even as T-ALL accounts for 15–20% of ALL cases. Among childhood cancer ALL is the most common and the third common leukemia through adults. Whereas ALL in children is more common than ALL in adults, childhood ALL has desirable prognosis and outcomes^1,2^. The complete remission rates are 85 to 90% and long-term survival rates are 30 to 50% through using intensive conventional chemotherapy regimen. ALL may have several relapses and may lead to dead^3,4^. Treatment among relapsed pediatric patients due to high toxicity regimen and low remission rates is not promising in second and subsequent relapses in following years of diagnosis^5^. Such an effective anti-leukemic potential and lower toxicity alternatives are necessary for these pretreated patients^6^.

Doxorubicin‐associated cardiotoxicity can occur during or after therapy and it leads to asymptomatic left ventricular (LV) dysfunction, cardiomyopathy, heart failure, and, in some cases cardiac death^7,8^. Despite researchers tried to improve strategies of leukemia treatment, their efforts have not yet achieved favorable outcomes. Relapse of the disease is still a major problem in ALL patients. Doxorubicin (Dox) from anthracyclin-antibiotic family is one of the most effective anti-tumor drugs, which has an important role in the first-line of ALL treatments^9^. Even though most of the patients with childhood leukemia are treated with Dox and they survive a long time after therapy. Many of them encounter a wide range of side effects such as cardiomyopathy and congestive heart failure, that it increased risk of sudden death^10^. In order to decrease the toxicity of Dox, physicians use limited dose and combined-modality strategies^11,12^.

In the last decades, various drugs are applied to treat the malignancies but some of them have a lot of undesirable effects and most of them are not efficient enough^13^. Accordingly, searching to find new drugs with fewer side effects is one of the most, if not the most, important aims in cancer therapies. Nowadays, plant extracts and natural products are the best choice in cancer therapy due to their fewer side effects^14^. One of the members of the Lamiaceae family is *Zataria Multiflora* (ZM) that is found in warm areas of Iran, Pakistan, and Afghanistan. ZM is used as spices to give foods odor and flavor^15^. In addition to its traditional applications, some studies indicate anti-spasmodic, antiseptic, and anesthetic effect of ZM extract (ZME). According to recent studies, researchers have demonstrated that ZME has various effects such as analgesic, anti-inflammatory, anti-nociceptive, anti-microbial and anti-oxidant properties^16–19^. Furthermore, its anti-tumor effect of ZME was investigated on some cancer cell lines^20^. In this study we assessed the apoptotic effect of ZME, Dox, and ZME/ Dox combination on Nalm-6 cells. ZME may potentiate the apoptotic effect of Dox in order to decrease its side effects. Moreover, present study aims to analyze the interaction between anti-apoptotic proteins and several main ingredients of ZME, as ligands, using docking simulations.

## Results

### ZME and DOX induce cell death on pre-B ALL cells

**Figure 1:**
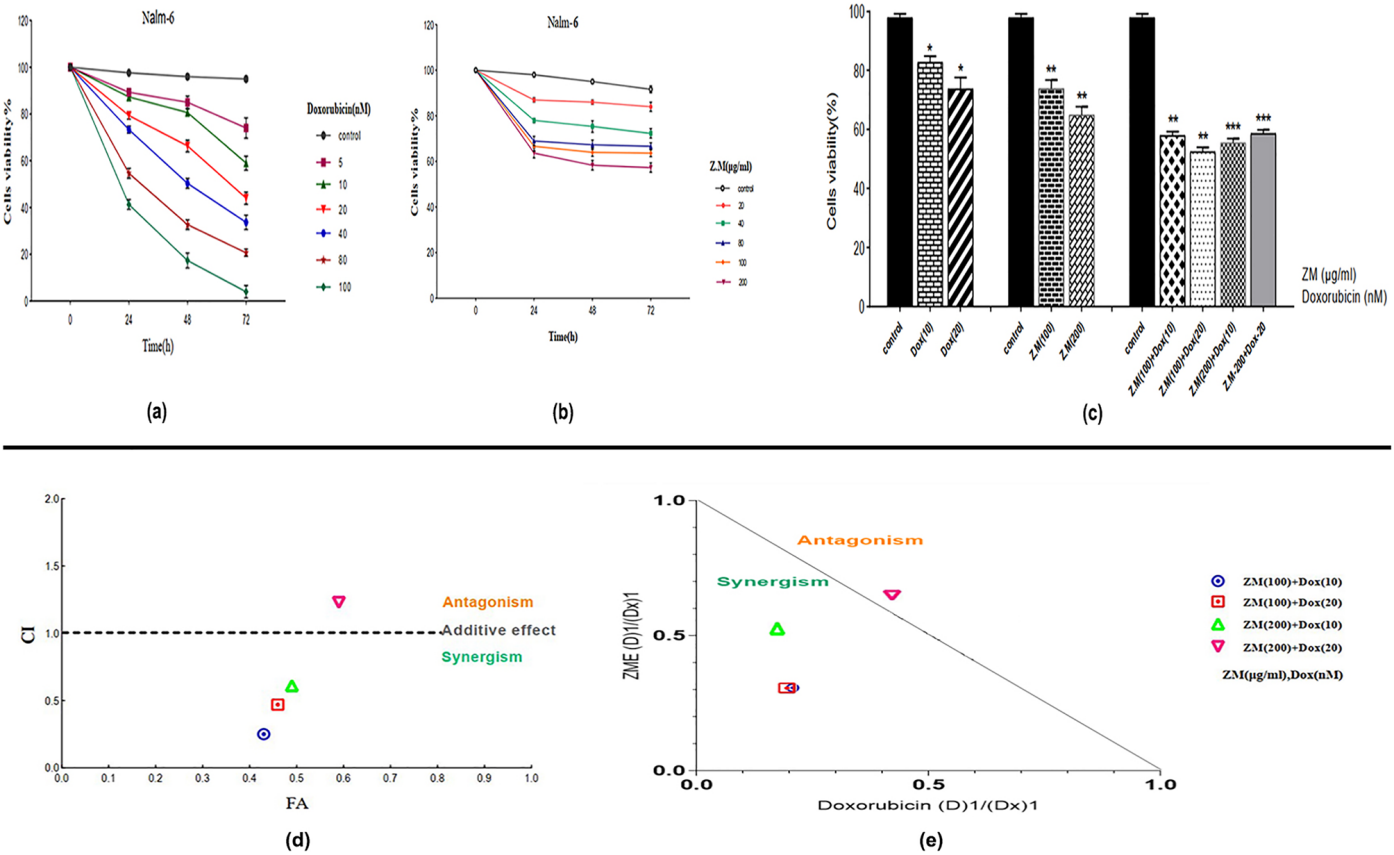
The ZME (**a**) and DOX (**b**) effect of various doses on the viability of Nalm-6 cells using trypan blue assay (mean ± SE, n = 3). These graphs present the changes of ZME and DOX apoptotic effect after 24, 48, 72 h. As shown, different concentrations of ZME and DOX had dose-dependent effect, dose and time-dependent effect, respectively. Effect of ZME and DOX combination and single doses (**c**) after 48 h on the viability of Nalm-6 cells using trypan blue exclusion assay (mean ± SE, n = 3). As shown, combination doses had a more significant apoptotic effect than individual doses on Nalm-6 cells (*P < 0.05, **P < 0.01, ***P < 0.001, relative to untreated cells). The combination index (CI) versus fraction effect (FA) curve (**d**) of ZME and DOX combination treatment. Exposure of Nalm-6 cells with various combinations of ZME and DOX and cell viability values of trypan blue assay was used for CI vs FA. As reported by FA curve, the CI < 1, = 1, and > 1 indicate respectively synergism, additive effect (solid line), and antagonism effect. Combinations of ZME (100 μg/mL) + DOX (10 nM) on Nalm-6 cells demonstrated the most desirable synergism effect among other combinations. Dose-normalized isobologram analysis of ZME and DOX combination. The CI was calculated according to the normalized isobologram equation (**e**). (Dx)1 and (Dx)2 indicate the individual dose of ZME and DOX required to inhibit a given level of viability index, and (D)1 and (D)2 are the doses of ZME and DOX necessary to produce the same effect in combination, respectively. Antagonism effect is represented by above points of the effect line, whereas the points are below the effect line demonstrate the synergism effect. Three combination points for Nalm-6 cells were below the effect line, so showed synergism effect.

**Figure 2:**
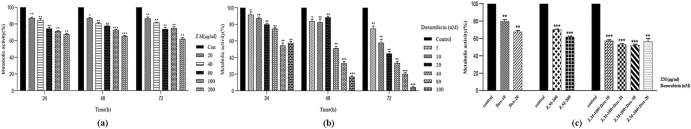
The effect of ZME (**a**) and DOX (**b**) on the metabolic activity of Nalm-6 cells 24, 48, 72 h after treatments. As shown, ZME had dose-dependent effect but DOX not only had dose-dependent effect but also had time-dependent effect. The effect of ZME and DOX combination against single doses after 48 h treatment on metabolic activity of Nalm-6 cells (**c**). The significant effect of drugs combination is visible in four doses (ZME and DOX: 100 + 10, 100 + 20, 200 + 10, 200 + 20) and we use ZME (100 μg/mL) and DOX (10 nM) as a combination dose for our study (*P < 0.05, **P < 0.01, ***P < 0.001, relative to untreated cells).

The viability percent of Nalm-6 cells treating with various concentrations of ZME and Dox individually and in combination were evaluated using trypan blue assay at 24, 48, 72 h after treatment (Fig. 1). The results demonstrated that the ZME and Dox decreased the viability of Nalm-6 cells. Dox had a dose- and time-dependent effect on cells, although ZME had only a dose-dependent effect. A significant decrease in viability of Nalm-6 cells was also seen in combination treatment. Here, interactions of the combination drug were evaluated using isobologram and combination index (CI) analyses. The isobologram analyses were conducted to assess the synergic effect of ZME and Dox on Nalm-6 cells. The CI-Fa curve (Fig. 1) indicated the synergistic effects (CI < 1) of all combination doses. As represented in Fig. 2, isobologram analysis indicated that all the points are below the line of additive effects in the synergism area. Moreover, the fraction-affect (FA) versus combination index analysis also demonstrated the synergistic (CI < 1) anti-proliferative effect of ZME/Dox combination on Nalm-6 cells (Table 1).

**Table 1:** CI and DRI for drug combination by ZME and DOX.

ZME (μg/mL)	DRI	Doxorubicin (nM)	DRI	CI value (At inhibition of 50%)
100	3.256	10	4.960	0.508
200	1.925	10	5.653	0.696
100	4.666	20	3.284	0.518
200	1.547	20	2.377	1.069

### ZME potentiated the cytotoxicity effect of DOX

Metabolic activity of treated cells with ZME and Dox was investigated with the MTT colorimetric method. As shown in Fig. 2a,b, ZME and Dox separately had cytotoxicity effect on Nalm-6 cells and they reduced the metabolic activity of treated Nalm-6 cells. Figure 2c presents the results of combination doses of ZME and Dox in which those are more efficient than individual doses. Outstandingly, the cytotoxic effect of Dox on Nalm-6 cells was potentiated by ZME using the synergistic combination treatments.

### ZME enhanced the effect of Dox on programmed cell death

**Figure 3:**
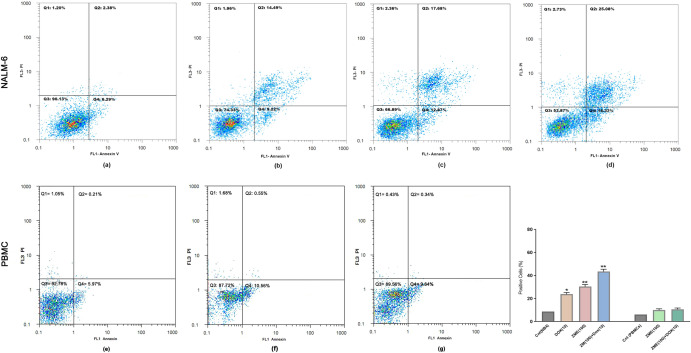
Nalm-6 cells were treated in selected combination and individuals: control (untreated) (**a**), DOX:10 nM (**b**), ZME:100 μg/mL (**c**), ZME: 100/DOX: 10 (**d**) for 48 h. Then, cells were analyzed for Annexin-V and Annexin-V plus Propidium Iodide (PI) by flow cytometry. As shown, ZME significantly enhanced the apoptotic effect of DOX on Nalm-6 cells in combination with more than an individual after 48 h treatment. As well, ZME has no significant apoptotic effect on PBMCs in determined combination (ZME: 100/DOX: 10) dose (**f**) and single (ZME: 100) dose (**g**) against the untreated control (**e**).

Here, 100 µg/mL ZME plus 10 nM Dox as a combination dose besides 100 µg/mL ZME and 10 nM Dox as individual doses were selected for investigation of Nalm-6 cells apoptosis. As shown in Fig. 3, the combination dose had a considerable increase in the percentage of Annexin-V and Annexin-V/PI positive cells. Consequently, a combination of 100 μg/mL ZME and 10 nM Dox induced 43.4% apoptosis, which is more than 23.71% apoptosis caused by 10 nM Dox alone (P < 0.0001).

### Inductive effect of ZME/Dox combination on the gene expression

**Figure 4:**
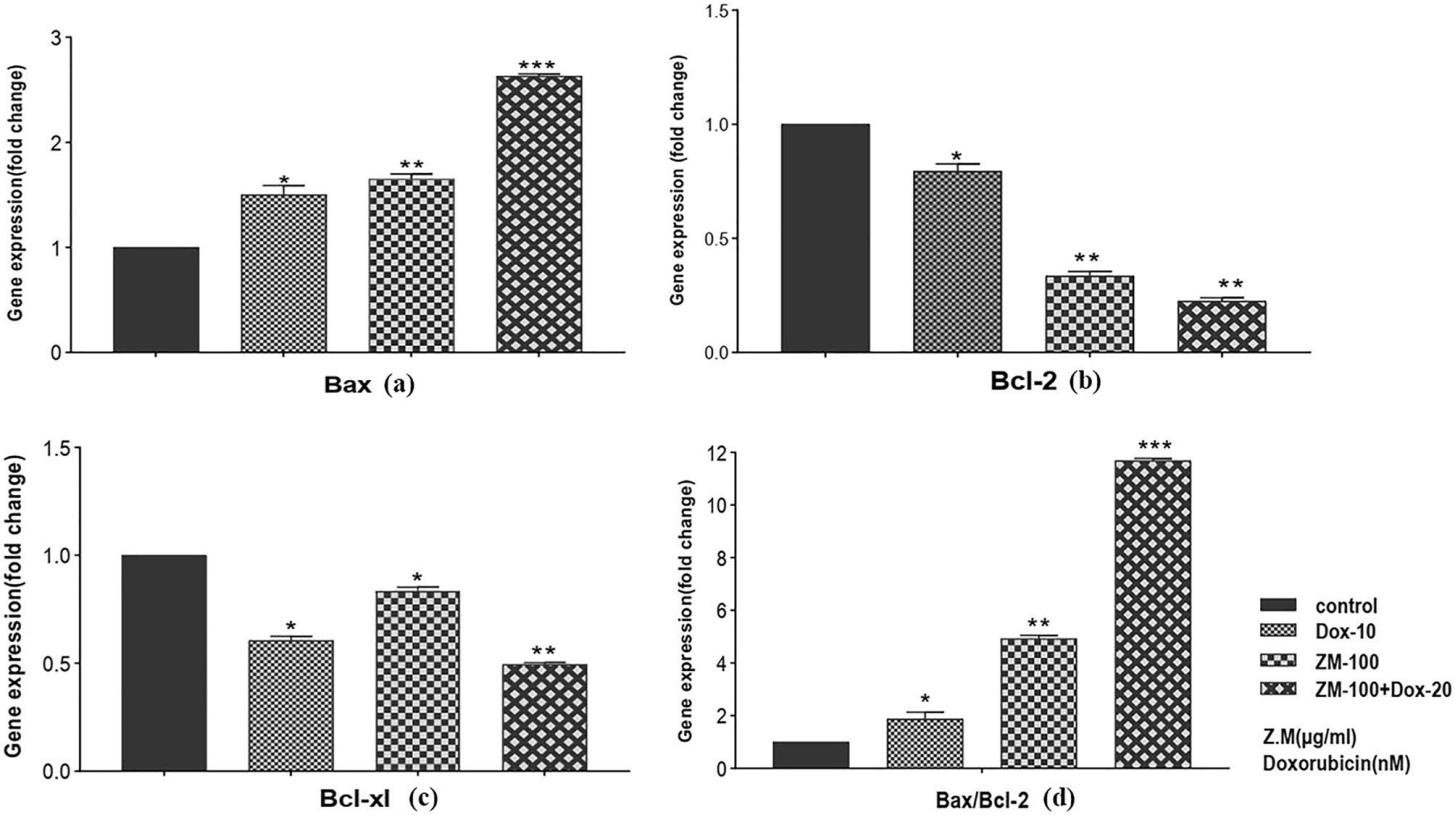
Fold change gene expression. Graph presents that ZME and DOX upregulate the *Bax* gene (pro-apoptotic) and down-regulates *Bcl-2* gene (anti-apoptotic). A combination dose of ZME and DOX changes gene expression more than single doses on Nalm-6 cells after 48 h treatment. *Bax* and *Bcl-2* ratio also was shown in this figure and demonstrated the significant effect of ZME and DOX in combination dose. In addition, *Bcl-Xl* from *Bcl-2* family was upregulated by ZME and DOX treatment in Nalm-6 cells after 48 h (*P < 0.05, **P < 0.01, ***P < 0.001, relative to untreated cells).

Nalm-6 cells were exposed to 100 μg/mL ZME, 10 nM Dox and a combination of them for 48 h. Thereupon, the expression of target genes was evaluated using quantitative real-time PCR. According to the results shown in Fig. 4**,** the expression of the *Bax* gene increased in the combination dose more than the single doses. These results also indicated the significant decreasing rate in *Bcl-2* gene expression which was influenced by the combination dose. Furthermore, *Bax* and *Bcl-2* expression ratio presented that the ZME enhances the Dox apoptotic effect on Nalm-6 cells (Fig. 4).

**Figure 5:**
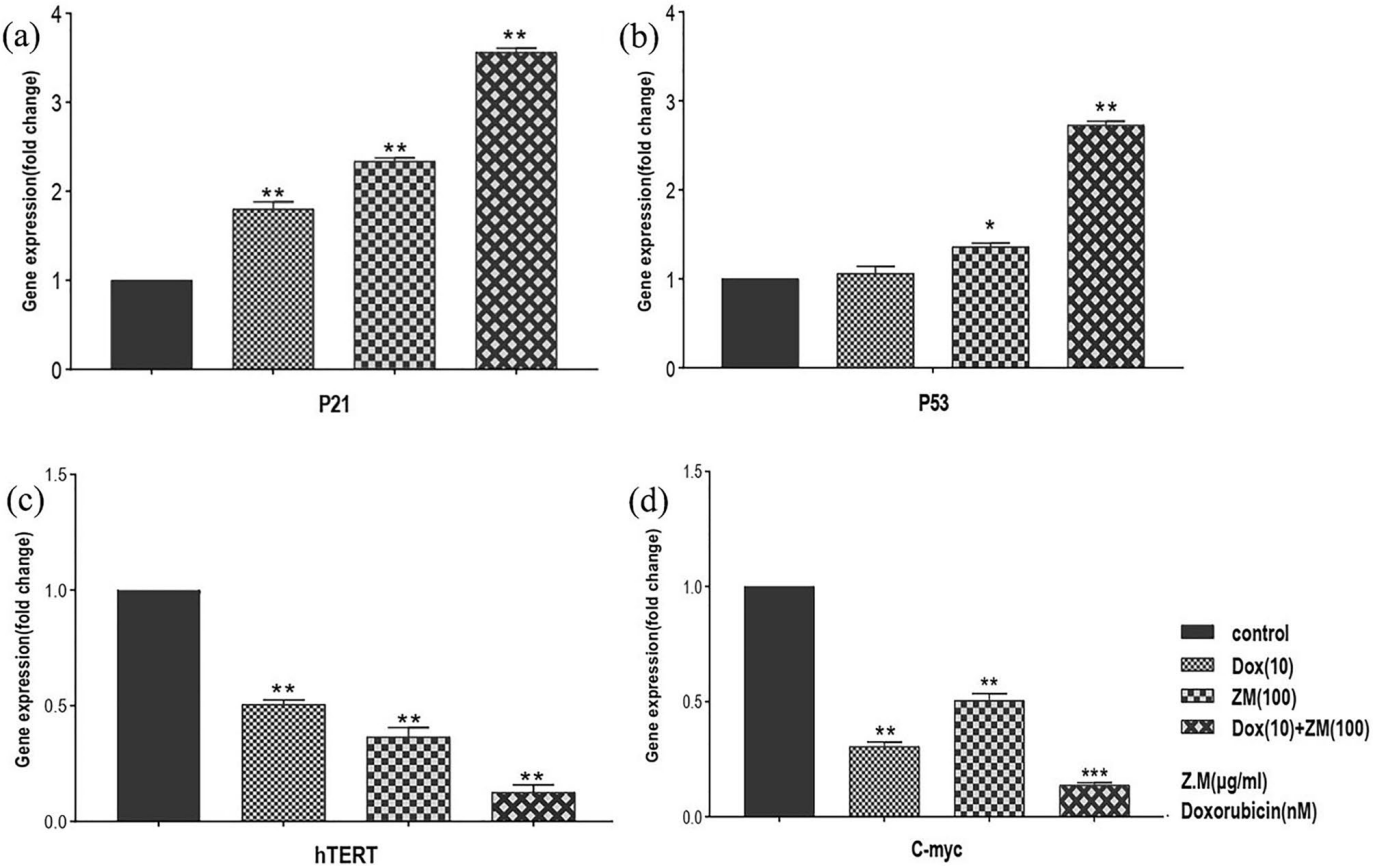
Gene expression graph presents that ZME and DOX down-regulates the h-TERT gene and *c-Myc* gene. A combination dose of ZME and DOX changes gene expression more than single doses on Nalm-6 cells after 48 h treatment. Fold change gene expression graph presents that ZME and DOX up-regulate *P21* gene and *P53* gene.

*P53* and *P21* were chosen as tumor suppressor genes to assess them in Nalm-6 cells treated with 100 μg/mL ZME, 10 nM Dox and combination of them after 48 h. As presented in Fig. 5a,b, the expression of the *P53* and *P21* genes was increased individually and combination dose of ZME/ Dox increased the gene expression more than single doses of ZME and Dox. As the results are shown in Fig. 5c,d, 100 μg/mL ZME, 10 nM Dox and a combination of them led to reduce the expression of *hTERT* and *c-Myc* genes. The combination dose of ZME/ Dox had more effective than ZME and Dox single doses.

### ZME had no significant effect on PBMCs

PBMCs were selected as a human normal cell to evaluate the effect of various concentrations of ZME. This study assessed the effect of ZME on PBMCs using MTT and flowcytometry assays after 48 h treatment. The results demonstrated that ZME had no significant impact on PBMCs metabolic activity. Moreover, according to Fig. 3, ZME treatments (single: 100 and combination: 100 + 10) have no noticeable effect on PBMCs.

### Molecular docking

**Figure 6:**
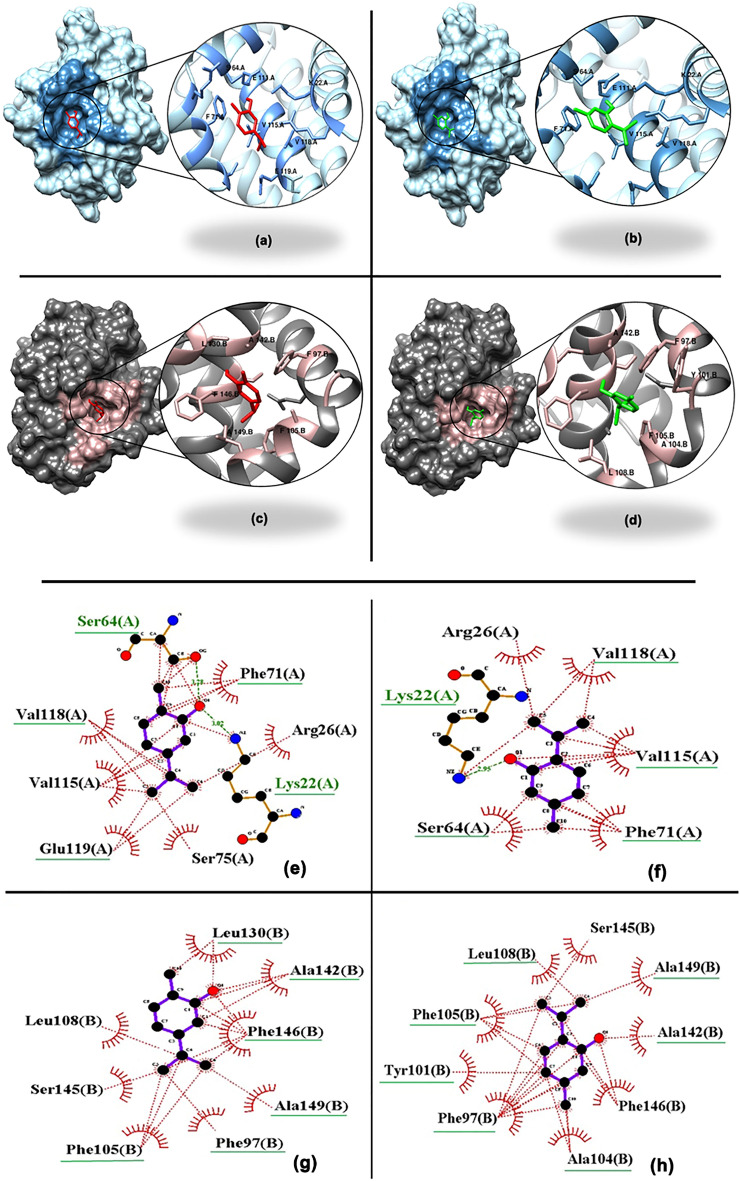
The 3D interaction of BCL2/carvacrol (**a**), BCL2/thymol (**b**), BCL-xl/carvacrol (**c**) and BCL-xl/thymol (**d**). The active site of BCL2 and BCL-xl are shown with dark blue and light pink, respectively. As well, carvacrol and thymol are seen in red and green color, respectively. Binding residues are presented in large scale circles. The 2D interaction of BCL2/carvacrol (**e**), BCL2/thymol (**f**), BCL-xl/carvacrol (**g**) and BCL-xl/thymol (**h**). The involved residues, which have bonds with macromolecules, are marked with green underline.

The ligand binding residues of *BCL2* and *BCL-xl* were computed by Moe active site finder. According to Moe active site of *BCL2* in chain A includes: LYS22, ARG26, ASP61, SER64, ARG65, ARG68, PHE71, ALA72, SER75, GLU111, GLY114, VAL115, VAL118, GLU119 and also the *BCL-xl* (chain B) active site for ligand binding includes: PHE97, TYR101, ALA104, PHE105, LEU108, GLN111, VAL126, GLU129, LEU130, ALA142, SER145, PHE146. Due to figure out the interaction among the structures and activities of our cytotoxic agents, molecular docking has been conducted under similar conditions via the AutoDock 4.2 tools program and MVD software. According to our result, thymol and carvacrol as ligands bind to the residues of *BCL2* and *BCL-xl* cavities with various bond and energy binding (Table 2 and Fig. 6). These interactions illustrates that these components could set the stage for the apoptosis pathway on Nalm-6 cells treatment with ZME and they may confirm the apoptotic effect of ZME on Nalm-6 cells.

**Table 2:** The optimal binding energy, hydrophobic interactions and hydrogen bonds of BCL2 and BCL-xl residues with carvacrol and thymol.

Ligands		Carvacrol	Thymol
BCL2	Hydrophobic Interactions	PHE71, VAL115, VAL118, GLU119	PHE71, VAL115, VAL118
	Hydrogen Bonds	LYS22, SER64, GLU111	LY22, SER64, GLU111
	Binding Energy (Mod Dock score)	− 47.51	− 52.69
	H Bond (kCal/mol)	− 3.38	− 3.56
BCL-xl	Hydrophobic Interactions	PHE97, PHE105, LEU130, PHE146, ALA149,	PHE97, TYR101, ALA104, PHE105, LEU108, ALA149
	Hydrogen Bonds	ALA142	ALA142
	Binding Energy (Mod Dock score)	− 65.01	− 65.69
	H Bond (kCal/mol)	− 1.93	− 2.53

## Discussion

With the increase of cancer incidence, many researches have been focused on new chemotherapeutic treatments^27^. One of the most useful and effective chemotherapeutic drugs is Dox which is used to treat wide groups of cancers, such as hematologic malignancies, leukemias, lymphomas and solid tumors like breast cancer. Todays, Dox is utilized widely in the chemotherapeutic regimens of cancer patients, even though Dox has a lot of side effects on patients^28,29^. Cardiotoxicity is one of the most important side effects of Dox causing cardiomyopathy, arrhythmia, congestive heart failure and finally heart failure in patients during or after treatment. To decrease the side effects, the dose of Dox should be reduced^30,31^. Nowadays, the cytotoxic effects of plant extracts on malignant cells are considered to use them as an alternative or complementary drug in chemotherapeutic treatment^32^. The plant extracts have low complications and they could be combined with the low doses of chemotherapeutic drugs in treatments to enhance the cytotoxic effect^33^. The results of this study indicated the cytotoxic effect of ZME individual and in combination with Dox on Nalm-6 cell line. Due to chemical composition analysis of *Zataria Multiflora* essential oil using GC/MS, recent studies have shown that the most ZME constituents are thymol, carvacrol, p-cymene and γ-terpinene, respectively^34–36^. The present study also evaluated the apoptotic effect of ZME and Dox individually and in combination on Nalm-6 cells. As shown in our results, the viability of Nalm-6 cells was reduced in exposure to ZME, Dox and their viability surprisingly was decreased in the combination doses more than those of individual. The results of Compusyn (Fig. 1), in addition, illustrated that three combination doses have significant synergistic apoptotic effect of ZME and Dox on the Nalm-6 cells (ZME and Dox: 100 + 10, 100 + 20, 200 + 10). Furthermore, it is clear in our results that ZME concentration doses potentiated low doses of Dox cytotoxicity effects on Nalm-6 cells. Owing to our results, we would rather choose 48 h incubation for combination investigation by flowcytometry. We, moreover, decided to select one dose before the IC50 doses of Dox and ZME for the combination therapy in order to reach the IC50 effect by combination treatment. So, Nalm-6 cells were treated with ZME: 100 µg/mL, Dox: 10 nM and combination of them to investigation the cytotoxic effect against untreated control. It is necessary to be mentioned that this study assessed the apoptotic effect of ZME and the combination dose with Dox (after 48 h incubation) on PBMCs. Due to flowcytometry results shown in Fig. 3, ZME concentration, individual and in combination, has no significant apoptotic effect on normal cells. In this regard, Janitermi el al. according to their MTT results, claimed that ZME has time- and dose-dependent cytotoxic effect on some cancer cell lines (MCF-7, AGS & HeLa), and it decreased the viability and metabolic activity of treated cancer cells. They also reported that ZME had no cytotoxicity effect on fibroblast cells as a normal cell^37–39^, and our MTT results, as well, indicate that there was no considerable cytotoxicity effect on PBMCs that were exposed to 100 and 200 µg/mL ZME. Additionally, according to recent studies, the apoptotic effect of ZME components like thymol, carvacrol, and P-Cymen was evaluated on malignant cell lines. Yi Li and colleagues reported that thymol downregulated the *Bcl-2* and *Bcl-xl* expression while it upregulated the *P21* expression in bladder cancer cell lines. They also showed that thymol did not have a cytotoxic effect on the urothelial cell line as non-malignant cell^40^. Recent researchers’ findings have shown that BCL-2 family proteins could be key regulators to control apoptosis mechanism. Some anti-apoptotic proteins such as BCL-2, BCL-xl and MCL-1 induce apoptosis process by binding to pro-apoptotic proteins. On the other hand, over expression of these anti-apoptotic proteins are breeding the ground for growth of several cancers by preventing apoptosis^41,42^. Fortunately, our flowcytometry and gene expression results illustrated that ZME provoked the apoptotic effect of Dox on Nalm-6 cells. The expression of *Bax* as we expected, has increased on treated cells with ZME and Dox. The *Bcl-2* expression was down-regulated on exposed cells with ZME and Dox. Moreover, hTERT is a central regulator of multiple hallmarks of various tumors, and the c-Myc and hTERT expression upregulate in most of the malignancies, and there is a correlation between them^43^. In this vein, our findings illustrate that the expression of c-Myc and *hTERT* reduced much more in combination dose of ZME and Dox than individual doses. In another experiment, Punia et al. also revealed that a combination of Dox and Acacetin (a plant derivative) enhanced the apoptotic effect on one type of lung carcinoma cells^44^. The increasing of *BCL2* as an anti-apoptotic factor in many cancers could be a promising target to combat malignant cells. Since *BCL2* inhibitors could occupy BH2 and BH3 positions and on the other hand according to our molecular docking analysis, carvacrol and thymol could interact with *BCL2* in BH3 and BH1 positions, they may inhibit *BCL2* to lead cancer cells to apoptosis pathway^45^. According to above explanations, since one strategy for cancers therapy is to find molecules that activate the cell death pathways. Hence, we analyzed the interactions of two high-concentration components of ZME with two of BCL2 family members (anti-apoptotic proteins) in the simulated space using molecular docking, and our molecular docking analysis demonstrated that carvacrol and thymol matched the molecules involving in apoptosis; so, they may trigger the apoptosis pathway. Furthermore, some recently studies utilized BCL2 and BCL-xl for docking simulations as anti-apoptotic proteins with several ligands^46,47^ and our findings also conform with them. Molecular docking studies and the biological results suggested that ZME can be a promising anticancer agent. In addition, these studies show the plant derivatives beside the alternative drugs and methods such using of nanoparticles, can act as drug complement in clinical therapies, particularly chemotherapeutic treatments^48,49^. Severe side effects are the most problem of chemotherapy drug, and due to attain a solution to eliminating the problem, researchers attempt to achieve alternatives or complements. Subsequently, the investigation of plant extracts on malignant cells is one of the best elections to induce the programmed cell death. As illustrated in the present study, ZME may be utilized as an alternative or a complement to decrease the effective dose of Dox for pre-B acute lymphoblastic leukemia cells.

## Material and methods

### Cell culture and materials’ preparation

Acute Lymphoblastic Leukemia cell line (Nalm-6, NCBI C212) was obtained from the Pasteur Institute collection, Tehran, Iran. Nalm-6 cells were cultured in RPMI-1640 with 2 mM l-glutamine (Gibco™ A1049101) containing 10% fetal bovine serum (FBS) (Gibco™ A3160402), 1% antibiotic (Penicillin–Streptomycin Solution 100X, Biowest, L0022) in a humidified atmosphere of 5% CO_2_ incubator at 37 °C. ZME was prepared according to methanolic extract protocol that Saedi Dezaki et al. applicated in their study^21^. ZME lyophilized powder was dissolved in dimethylsulfoxide (DMSO) (Merck, CAS 67-68-5) and culture media as main ZME stock, and diverse concentrations of ZME were diluted and obtained from main solution stock. The stock of doxorubicin (EBEWE Pharma, Austria) was also diluted into considered concentration for treatment.

### Trypan Blue assay

To evaluate the apoptotic effects of ZME and Dox on cell viability, Nalm-6 cells (250 × 10^5^ cells/mL) were seeded in 12-well plate and incubated in the presence of the various concentrations of ZME (20, 40, 80, 100, 200 µg/mL) and Dox (5, 10, 20, 40, 80,100 nM) individually for 24, 48, and 72 h. After that, the cell suspension was centrifuged and the cell pellet was suspended in a serum-free complete medium. Next, one part of 0.4% trypan blue (Gibco™ 15250061) and one part of cell suspension was mixed and then allowed mixture to incubate 2 min at room temperature. The total number of unstained (viable) and stained (non-viable) cells was manually counted by Neubauer chamber and light microscope (ECLIPSE E100, Nikon). Finally, the percentage of viable cells was calculated as “Viability (%) = viable cells/viable cells + death cells × 100”^22^.

### Determination of combination index and dose reduction index

To estimate the interaction between ZME and Dox, the combination index (CI) was calculated using CompuSyn Software (ComboSyn, Inc., Paramus, NJ, USA) according to the classic isobologram equation: “CI = (D)_1_/(Dx)_1_ + (D)_2_/(Dx)_2_”, where (Dx)_1_ and (Dx)_2_ represent the individual dose of ZME and Dox required to inhibit a given level of viability index, and (D)_1_ and (D)_2_ are the doses of ZME and Dox necessary to produce the same effect in combination, respectively. Since different CI values (< 1, = 1, > 1 indicate synergism, additive effect, and antagonism, respectively) can be observed at different levels of growth inhibition (fraction affected, FA), CI versus FA plots were applied to present the data using MS Excel. The dose which may be decreased in a combination for a given level of effect as compared to the concentration of individual drug alone defined as dose reduction index (DRI) and calculated as follow: (DRI)_1_ = (Dx)_1_/(D)_1_ and (DRI)_2_ = (Dx)_2_/(D)_2_^23,24^.

### MTT assay

In vitro screening of the cytotoxicity effect of ZME and Dox towards cancer cell lines was measured using MTT colorimetric assay. The metabolization of thiazolyl blue tetrazolium bromide into formazan crystals by Nalm-6 alive cells was assessed by this test. Hence, 1 × 10^4^ Nalm-6 cells were seeded in 96-well plates with various concentrations of ZME (20, 40, 80, 100, 200 µg/mL) and Dox (5, 10, 20, 40, 80,100 nM) individually for 24, 48, and 72 h. Afterward, the plate was centrifuged at 700×*g* for 10 min and the supernatant was removed. The cells were incubated with 100 µL MTT solution (0.5 mg/mL; (M5655, Sigma) at 37 °C. After 4 h, colored formazan was solubilized by the addition of 150 μL DMSO at each well and optical absorbance was evaluated at 570 nm with an enzyme-linked immunosorbent assay reader. The percentage of metabolic activity of treated cells was calculated relative to untreated cells which were set as negative control. In addition, MTT test was performed for combination dose of ZME/ Dox (100 µg/mL ZME + 10 nM Dox, 100 µg/mL ZME + 20 nM Dox, 200 µg/mL ZME + 10 nM Dox, 200 µg/mL ZME + 20 nM Dox). In addition to untreated cells, Nalm-6 were treated with the highest concentrations of DMSO (were used in our study) as a negative control owing to use it for dissolving ZME (0.01% and 0.1%).

### Flowcytometry

The flowcytometry technique was used to assess the effect of ZME and Dox on the induction of early and late apoptosis using annexin V-propidium iodide (PI) staining. Consequently, 4 × 10^5^ Nalm-6 cells were seeded into six-well cell culture plates and treated with ZME (100 µg/mL), Dox (10 nM) and combination (100 µg/mL ZME and 10 nM Dox). Then, after 48 h, the cells were collected and they were washed with PBS. Flowcytometry was performed using Annexin-V Apoptosis Detection Kit (Mab Tag, AnxF100PI) and the results were analyzed using the FlowJo.7.6.1 software.

### RNA isolation and preparation of cDNA

YTzol Pure RNA (Yekta Tajhiz Azma, YT9066) was used to isolate total RNA from untreated (control) and treated cells with 100 µg/mL ZME, 10 nM Dox and ZME/ Dox combination (100 µg/mL ZME and 10 nM Dox). Quantity of RNA samples was assessed by NanoDrop (NanoDrop ND-1000; Thermo Scientific, Wilmington, DE) at A260/A280 ratio. The quality and purity of extracted RNA were illustrated by agarose gel electrophoresis. Reverse transcription (RT) reaction was carried out according to the manufacturer instructions using the RevertAid First Strand cDNA Synthesis kit (Thermo Scientific Fermentas, K1622).

### Quantitative Real-time PCR

Changes in mRNA expression of desired genes were surveyed by real-time PCR. Quantitative real-time PCR was performed by 10 μL containing Real Q Plus 2 × Master Mix Green (Amplicon, Denmark, A325402), 1.5 μL of the cDNA product, 1 μL of forward and reverse primers (10 pmol of each other), and 7.5 μL of nuclease-free water. Thermal cycling conditions included an initial activation step at 95 °C for 15 min followed by 40 cycles, a denaturation step at 95 °C for 15 s and a combined annealing/elongation step at 60 °C for 60 s. The reaction took place in the RotorGene® Q Real-time PCR System (Qiagen, USA). A melting curve analysis was performed to verify the specificity of the products. The fold change was measured relative to the control and calculated after adjusting for the B-actin reference gene using Ct (2^−ΔΔCT^) method. Nucleotide sequences of the primers used for real-time RT-PCR listed in Table 3.

**Table 3:** Primer sequences used for quantitative real-time PCR.

Gene	Forward primer	Reverse primer
B-actin	5’-CCAACCGCGAGAAGATGA-3’	5’-TCCATCACGATGCCAGTG-3’
hTERT	5'- CGGAAGAGTGTCTGGAGCAA -3'	5'- GGATGAAGCGGAGTCTGGA -3'
c-MYC	5'-GTCCTCGGATTCTCTGCTCTC-3'	5'-CAACATCGATTTCTTCCTCATCTTC-3'
P21	5'-CCTGTCACTGTCTTGTACCCT-3'	GCGTTTGGAGTGGTAGAAATCT-3'
P53	5'-CTGGCCCCTGTCATCTTCTG-3'	5'-CCGTCATGTGCTGTGACTGC-3'
Bax	5’-AGGATCGAGCAGGGCGAATG-3’	5’-TCAGCTTCTTGGTGGACGCA-3’
Bcl-2	5’-ATCGCCCTGTGGATGACTGAG-3’	5’-CAGCCAGGAGAAATCAAACAGAG-3’
Bcl-xl	5’-TGCATTGTTCCCATAGAGTTCCA-3’	5’-CCTGAATGACCACCTAGAGCCTT-3’

### ZME effect on normal cells

Peripheral blood mononuclear cells (PBMCs) were isolated from healthy donor using density gradient centrifugation using Ficoll-Hypaque density gradient (Lymphodex, Germany). Isolated cells were washed two times by PBS. Thus, the pellet was resuspended in 1 mL complete media (containing RPMI-1640 with 2 mM l-glutamine, 10% FBS and 1% antibiotic) and cultured in 8 mL complete media at the same condition used for Nalm-6 cells. These cells were treated with ZME (100 and 200 µg/mL) and combination (ZME: 100 µg/mL with Dox: 10 nM) and incubated at 37 °C so as to assess metabolic activity after 48 h using MTT. PBMCs, as well, were treated with 100 µg/mL ZME and combination dose (100 µg/mL ZME and 10 nM Dox) and measure the apoptosis rate after 48 h by flowcytometry.

### Molecular docking

The 3D structure of antiapoptotic proteins such as BCL2 (PDB ID: 2W3L)^25^ and BCL-xl (PDB ID: 2YXJ)^26^ were obtained from the RCSB protein data bank (https://www.rcsb.org). In addition, the ligands of these complexes were eliminated using the software MOE 2019.102 (Molecular Operating Environment) to prepare the macromolecules for docking stimulation. Thymol (CID:6989), carvacrol (CID:10364) structures were retrieved from PubChem compound database (https://pubchem.ncbi.nlm.nih.gov/). MOE was used to compute and find the active sites of *BCL2* and *BCL-xl* for ligands binding. Here, AutoDock Tools 4.2 and MVD software (Molegro Virtual Docker 6.0.1) have been applied for molecular docking. The active sites were input and the grid box dimension has been adjusted to optimized size surrounding the active site of the protein in order to ensure the free rotation of the ligands in the inner side of the grid. The docking run numbers have been estimated to be 100. The resulting poses have been chosen according to the corresponding binding energy. Using LigPlot analysis, the interactions of ligands and involved amino acids of proteins were analyzed, and the results depict in 2D interaction between ligands and macromolecules. Ultimately, the 3D shapes of the final interaction were drawn using the software Chimera 1.12 to clarify the interactions.

### Statistical analysis

Experimental data are expressed by mean ± standard deviation (SD) to compare the mean values among experimental groups using SPSS version 18.0 (SPSS, Inc., Chicago, IL, USA). All tests were done in duplicate or triplicate. Statistical analysis of MTT and Trypan blue data was calculated by Two-way ANOVA test and One-way ANOVA analysis was used to evaluate the data of flowcytometry and Quantitative real-time PCR. Statistically different values were defined significant at *p ≤ 0.05, **p ≤ 0.01 and ***p ≤ 0.001.

### Ethics statements

This study was approved by Kerman University of Medical Science Ethical & Research Committee (ethical code: IR.KMU.REC.1398.397). The volunteers were informed about the objective and procedure of the study and those who were willing to participate, donate blood sample and sign an informed consent were recruited. All methods were performed in accordance with relevant guidelines and applicable regulations.

## Data Availability

The 3D structure of proteins which used in molecular docking were obtained from Protein Data Bank (https://www.rcsb.org), and the PDB ID of BCL2 and BCL-xl, according to this data bank, are 2W3L (https://www.rcsb.org/structure/2W3L) and 2YXJ (https://www.rcsb.org/structure/2YXJ), respectively. The 3D structures of our ligands were obtained from PubChem compound database (https://pubchem.ncbi.nlm.nih.gov/). The ID of Thymol and carvacrol are respectively CID:6989 (https://pubchem.ncbi.nlm.nih.gov/compound/6989), CID:10,364 (https://pubchem.ncbi.nlm.nih.gov/#query=10364).
